# The Keystone Veterinary Medical Association

**Published:** 1901-07

**Authors:** E. M. Ranck

**Affiliations:** Secretary


Secretary.

---

### THE KEYSTONE VETERINARY MEDICAL ASSOCIATION.

### APRIL.

THE monthly meeting was held at Broad and Filbert Streets, Philadelphia, Pa., April 9, 1901, with the following members of the profession present: Drs. Lintz, Ridge, T. B. Rayner, Underhill, Schneider, Carter, Marshall, Rhoads, and Ranck.

Dr. Underhill read an act to regulate the practice of veterinary medicine and dentistry in Iowa.

Dr. Ridge reported a case of a grade Jersey cow which stopped eating but showed no other symptoms at first. Gave her tonics but deferred diagnosis; lungs and heart all right; constipated, and feces fetid. Gave her sulphate of soda, which partially overcame the constipation, but feces remained fetid. Temperature, 99° to 100°. The stable-man said she did not urinate, so the doctor catheterized her, and drew one-half pint of chocolate-colored urine; noticed violent straining, and discharged a large clot of blood three-eighths inches in diameter, presumably from the urethra, very tenacious and rope-like. Has not as yet made positive diagnosis.

Dr. Ranck reported a case of a rash on a dog which seemed to resemble mange, though the microscope revealed nothing of the insects, but on which he was applying epicarin and sweet-oil, one-half ounce to two ounces, but had not progressed far enough for final decision as to the effect of the epicarin.

Dr. Carter reported good results in these conditions from the hyposulphite of soda treatment—internal and external.

Dr. Ridge said the best remedy for skin diseases, especially in horses and cows, was a preparation of equal parts of crude pyroligneous acid, linseed-oil, and oil of turpentine, with salines internally.

Dr. Schneider reported a case of rabies, the diagnosis of which was verified by experiments made by Dr. Ravenel.

Dr. Lintz reported several cases which resembled rabies, but which, after being kept in quarantine for a certain length of time, proved to be some other nervous condition, and which ordinarily would have been killed had not the proper measures been taken.

Dr. Rayner reported a new method of castration of dogs, which was unique and effective when large animals have to be castrated without assistance.

Dr. Rhoads reported a case he heard of in which a poker was used as an aphrodisiac; also a case of a valuable collie dog eight or nine months old which was very weak across the loins. No history of being hurt, but is not yielding to treatment. He is giving Sanmetto and motor stimulants.

The meeting adjourned for lunch.

E. M. Ranck,
Secretary.

